# CO_2_ Capture with Polyethylenimine Supported on 3D-Printed Porous SiO_2_ Structures

**DOI:** 10.3390/ma17122913

**Published:** 2024-06-14

**Authors:** René Wick-Joliat, Florian B. Weisshar, Michal Gorbar, Daniel M. Meier, Dirk Penner

**Affiliations:** IMPE Institute of Materials and Process Engineering, School of Engineering, ZHAW Zurich University of Applied Sciences, Technikumstrasse 9, 8401 Winterthur, Switzerland; renewick@gmail.com (R.W.-J.); florianbalthasar.weisshar@zhaw.ch (F.B.W.); michal.gorbar@zhaw.ch (M.G.); danielmatthias.meier@zhaw.ch (D.M.M.)

**Keywords:** CO_2_ capture, material extrusion 3D printing, fumed silica, polyethylenimine, pressure drop

## Abstract

Amines supported on porous solid materials have a high CO_2_ adsorption capacity and low regeneration temperature. However, the high amine load on such substrates and the substrate itself may lead to substantial pressure drop across the reactor. Herein, we compare the CO_2_ adsorption capacity and pressure drop of fumed silica powder to 3D-printed monolithic fumed silica structures, both functionalized by polyethylenimine (PEI), and find a drastically reduced pressure drop for 3D-printed substrates (0.01 bar vs. 0.76 bar) in the sorption bed with equal CO_2_ adsorption capacity. Furthermore, the effect of 3D-printing nozzle diameter and PEI loading on the adsorption capacity are investigated and the highest capacities (2.0 mmol/g at 25 °C with 5000 ppm CO_2_) are achieved with 0.4 mm nozzle size and 34 wt% PEI loading. These high capacities are achieved since the 3D printing and subsequent sintering (700 °C) of monolithic samples does not compromise the surface area of the fumed silica. Finally, the comparison between 3D-printed monoliths and extruded granulate of varying diameter reveals that the ordered channel system of 3D-printed structures is superior to randomly oriented granulate in terms of CO_2_ adsorption capacity.

## 1. Introduction

With CO_2_ levels rising at unimpeded speed since the start of the Industrial Revolution, carbon capture and storage (CCS) has become a key field of research in the last two decades.

Amines supported on various high-surface-area solids have shown promising CO_2_ adsorption capacities of up to 5 mmol/g, as highlighted in a recent review [1]. While tetraethylenepentamine (TEPA) features the highest capacities [2,3,4], polymeric amines such as polyethylenimine (PEI) have emerged in recent years due to their superior stability and oxidation resistance [5,6]. Further studies have developed strategies to decrease the regeneration temperature of PEI-impregnated silica [7,8].

Economical, large-scale CO_2_ capture also requires a low pressure drop across the sorbent reactor. The 3D printing of the solid sorbent has emerged as an attractive method to achieve low pressure drops and high CO_2_ capacities due to the combination of macrochannels induced by the 3D printing and the hierarchical porosity of the materials. This strategy was tested extensively for MOF and zeolite substrates, with promising results [9,10,11,12]. In comparison, 3D-printed silica substrates are underrepresented in the literature. Thakkar et al. investigated the direct ink writing (DIW) of silica loaded with PEI or TEPA [13]. They either printed the silica first and functionalized the substrate after sintering or directly printed the PEI-functionalized silica and performed CO_2_ adsorption tests without sintering the substrate. Sluijter et al. used a direct light processing (DLP) printer to fabricate porous cylindrical monoliths and functionalized them after sintering with trimethoxy silyl propyl ethylenediamine. However, their substrate lost most of the surface area during sintering, leading to much lower CO_2_ capacities in the 3D-printed samples as compared to functionalized silica powder [14]. A computational study by Krishnamurthy et al. showed a 2.35-times improvement for 3D-printed vs. packed-bed sorbent (amino silane-functionalized silica) [15].

Here, we present a strategy to fabricate fumed silica monoliths by material extrusion (MEX) 3D printing with no surface area loss after sintering as compared to the raw powder. In MEX (previously called fused deposition modeling (FDM) or fused filament fabrication (FFF)), a molten thermoplastic material is extruded through a nozzle and deposited layer-wise on a printing bed to form three-dimensional objects. The method is also applied for metal or ceramic 3D printing by filling a thermoplastic material with high contents of metallic or ceramic powder (approx. 50 vol%) [16,17,18,19,20,21,22].

In this report, the so-called feedstock is prepared by mixing fumed silica powder with an organic binder system. Such binders typically consist of a backbone polymer responsible for the mechanical strength of the printed samples, water-soluble auxiliary binder facilitating debinding and sintering, as well as surfactants and dispersants to both optimize the interaction between silica particles and binder polymers and adjust the rheology of the feedstock. A binder system used for commercial ceramic injection molding (CIM) is used as a starting point for feedstock development, since most feedstock requirements are shared between CIM and ceramic MEX 3D printing. MEX offers the advantages of low investment and running costs, simplicity and comparatively high speed, at the cost of low resolution. Silica cylinders are printed with a triply periodic minimal surface (TPMS)-type gyroid structure, which has been shown to be superior to other regular 3D structures for a wide range of applications, like catalysis, heat exchange or adsorption [23,24,25]. After debinding and sintering, the silica cylinders are functionalized with branched PEI with a molecular weight of 10,000 Da. This PEI is chosen because it offers significant stability advantages over lower-molecular-weight PEI (600–1200 Da), albeit at the cost of lower CO_2_ adsorption capacities [26,27].

Finally, silica monoliths 3D printed with different nozzle sizes and sintered at different temperatures are characterized and the CO_2_ adsorption capacity and pressure drop between 3D-printed substrates and powders are compared.

## 2. Materials and Methods

### 2.1. Materials

Fumed silica (S5505), branched polyethylenimine (PEI, average MW: 10,000 Da) and polyethylene glycol (PEG, average MW: 400 Da) were purchased from Sigma-Aldrich, the binder system SW BS from EnCeram (Budenheim, Germany), isopropanol (purum) from Hänseler AG (Herisau, Switzerland) and ethanol (absolute) from Alcosuisse (Büren, Switzerland).

### 2.2. Feedstock Preparation

Firstly, the water-soluble fraction of the EnCeram SW BS binder system was removed and replaced by shorter-chain-length PEG to optimize the rheology of the feedstock. Therefore, the binder powder was suspended in water for 48 h to leach out the water-soluble part. The insoluble residue was filtrated and rinsed with water and dried at 40 °C for 24 h. The binder particles in the range of 250–500 µm were collected via sieving for further feedstock preparation. 

A silica suspension was prepared by adding 43.4 g fumed silica powder to 100 mL isopropanol and homogenizing the suspension in a DAC 150 SpeedMixer by Hauschild (Hamm, Germany) at 2000 rpm for 30 s. In the next step, 28 g EnCeram binder (with the water-soluble fraction previously removed) and 6.1 g PEG 400 were added before speedmixing again at 2000 rpm for 2 × 30 s. The resulting paste was fed into a twin-screw extruder (Thermo Fisher (Waltham, MA, USA), Process11 Extruder) and processed at 130 °C and 120 rpm. For the first extrusion cycle, most isopropanol evaporated and therefore the nozzle was removed from the extruder. The feedstock was extruded as white powder, which was extruded again, this time through a 1.5 mm nozzle, and 5 g additional EnCeram SW binder was added to the mixture before the next extrusion cycle. In this way, 5 g binder was added after each extrusion cycle until a soft and smooth filament was extruded containing a total of 70 g EnCeram binder. The extruded filament was granulated and extruded two more times. Finally, the filament was granulated and sieved to particle sizes of 200–1000 µm for 3D printing.

### 2.3. Printing

All parts were printed on a multimaterial MEX printer constructed at ZHAW (Winterthur, Switzerland). The printer features three print heads, two for granulated feedstock and one for filaments. Details about the printer can be found in a previous report [28]. For this study, only one of the granulate print heads was used. Relevant printing parameters are listed in Table 1. The 3D-printed cylinders used for CO_2_ capture were 20 mm in height and 10 mm in diameter. The parts were sliced in the Simplify 3D software V 5.0 using the gyroid infill at 50% density without perimeter lines and without top/bottom layers.

### 2.4. Filament Extrusion

The MEX printer was also applied to extrude continuous feedstock filaments with the same nozzle diameters as used for 3D printing. For each nozzle size (0.4, 0.6, 0.8 mm), 3–4 g of filament was extruded and the filaments were debound and sintered together with the 3D-printed samples.

### 2.5. Debinding/Sintering

**Figure 1:**
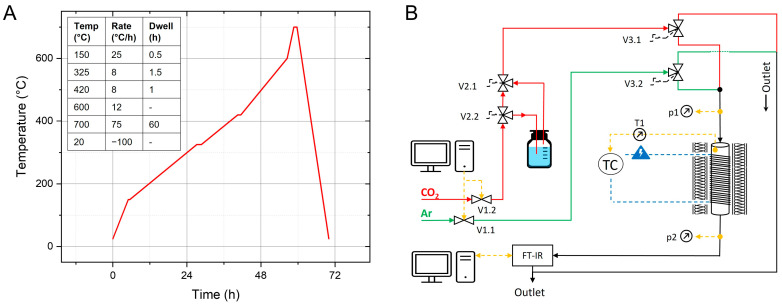
(**A**) Sintering profile for SiO_2_ samples; (**B**) setup for CO_2_ adsorption measurements.

After printing, the parts were immersed in a deionized water bath for 24 h to leach out the PEG. After drying for 24 h at 40 °C the samples were thermally debound and sintered in an ashing furnace (AAF 11/7, Carbolite Gero GmbH & Co. KG, Neuhausen, Germany) according to the sintering profile shown in Figure 1A. Some samples were sintered at 800 or 900 °C, as mentioned in Section 3.3.

### 2.6. Characterization (Microscopy, SEM, BET, Compression Strength)

Sintered samples were characterized by light microscopy (VHX 6000, Keyence, Osaka, Japan) and scanning electron microscopy (Phenom XL Desktop, Thermo Fisher Scientific, Waltham, MA, USA). Gas adsorption measurements and specific surface area calculations according to BET model were performed on a NOVA 2000e (Quantachrome, Boynton Beach, FL, USA) with nitrogen gas and five measurement points. Prior to the measurements, the samples were dried at 150 °C under vacuum for 1 h. Surface areas are reported as the average of three to four measurements of different samples and the corresponding standard deviation. Compression strength tests were conducted on a Zwick universal testing machine (Z010, ZwickRoell, Ulm, Germany) with a testing speed of 0.2 mm/min on 3D-printed and sintered cylinders (0.4 mm nozzle size, 50% gyroid infill) with 10 mm diameter and 10 mm height. 

### 2.7. PEI Surface Modification

Firstly, a stock solution of polyethylenimine (PEI) was prepared by dissolving 1 g of PEI in 10 g of ethanol. This stock solution was then added dropwise onto sintered silica cylinders (unmodified weight 0.33–0.40 g) until the whole cylinder was soaked (0.5–0.7 mL). The samples were then dried at 40 °C for one hour and 90 °C for an additional hour. Higher PEI loadings were achieved by repeating this process up to five times. 

PEI modification of silica powder and filament granules was achieved by wet impregnation. PEI according to the resulting loading amount of the final product was dissolved in ethanol in a sonication bath. After complete dissolution, the PEI solution was added to the powder/filament and mixed in a rotavapor for 15 min at 50 °C. Then, the solvent was removed under reduced pressure at 50 °C. 

### 2.8. CO_2_ Adsorption 

The CO_2_ adsorption experiments were performed in a self-built tube reactor (ø ID: 10 mm) with electrical heating, as shown in Figure 1B. For each measurement, 500–600 mg of powder and granules were placed on top of a bed of glass wool inside the tube reactor. The 3D-printed and functionalized cylinders (400–680 mg) were surrounded with ceramic paper (ProTherm GmbH, Wangen-Brüttisellen, Switzerland) to fit them tightly inside the reactor tube. Before measuring the adsorption capacity, the samples were conditioned and heated 2.5 °C/min to 100 °C under constant volumetric argon flow (200 mL/min). This temperature was maintained for 10 min and then the reactor was cooled to 25 °C. Subsequently, the gas was switched to a 5000 ppm CO_2_ in N_2_ (200 mL/min) gas stream, which was humidified by passing it through a gas washing bottle filled with deionized water. The outlet CO_2_ concentration was monitored by online Fourier-transform infrared (FTIR) spectroscopy [ν(CO_2_) = 2358 cm^−1^] (Bruker ALPHA, Bruker, MA, USA). For the pressure drop measurements, the 3D-printed cylinders (sealed with ceramic paper) were stacked, or the corresponding mass of 30% PEI on SiO_2_ powder on a bed of glass wool was placed in the reactor and a constant volumetric argon flow (200 mL/min) was set. The resulting pressure difference directly before and after the reactor tube was measured by two digital pressure sensors (Swagelok Transducer S model, Swagelok, Solon, OH, USA).

## 3. Results and Discussion

### 3.1. Feedstock

The feedstock used for MEX 3D printing was prepared from fumed silica powder and a CIM binder system. The ingredients underwent multiple rounds of mixing via extrusion using a twin-screw extruder. A high volume fraction of fumed silica in the feedstock is necessary to avoid cracking and deformations during debinding/sintering. On the other hand, a high content of binder ensures sufficient flowability for reliable and defect-free 3D printing. Therefore, feedstock development started with a 40/60 vol% mixture of silica and binder and the binder content was gradually increased until a soft and smooth feedstock was extruded at 22.3 vol% fumed silica and 77.7 vol% binder.

### 3.2. Three-Dimensional Printing

**Figure 2:**
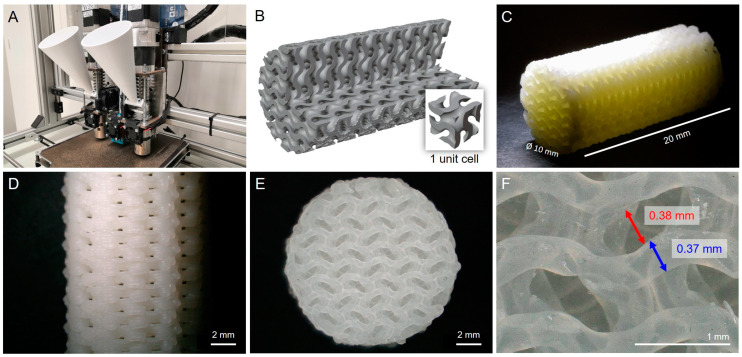
(**A**) Multimaterial MEX 3D printer used in this work. (**B**) CAD designs of gyroid structures with 50% density. (**C**–**F**) Light microscopy images of a SiO_2_ cylinder printed with a 0.4 mm nozzle before sintering.

All parts were printed on a granulate-fed MEX printer, which was designed and constructed at ZHAW (Figure 2A). A cylinder of 20 mm length and 10 mm diameter was chosen as the standard sample size, because those cylinders would fit into the CO_2_ adsorption measurement setup. The cylinders were printed with a gyroid infill at 50% density (Figure 2B). Consequently, the wall thickness was one printing line, corresponding roughly to the nozzle diameter, and the open channel width was approximately the same as the wall thickness (Figure 2C–F). In general, TPMS architectures such as the selected gyroid structure are known for a favorable combination of mechanical strength and low pressure loss [29].

### 3.3. Sintering

**Figure 3:**
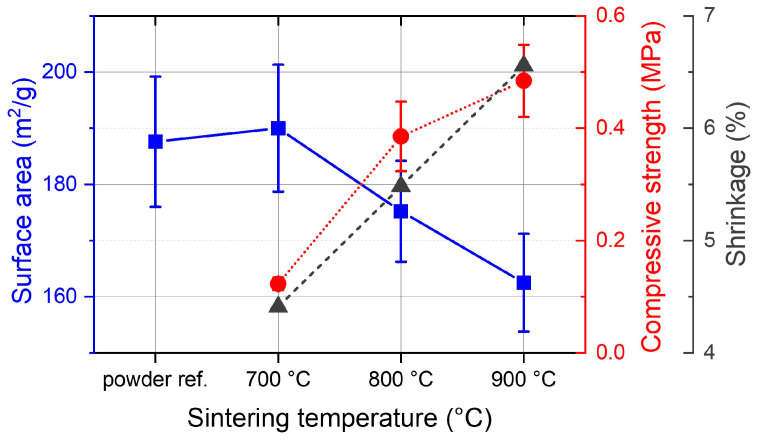
BET surface area (left axis, blue squares), compressive strength (right axis, red circles) and shrinkage (right axis, black triangles) of fumed silica powder and 3D-printed parts sintered at different temperatures.

After printing, the gyroid cylinders were immersed in a water bath to dissolve the 10 vol% of PEG from the material. During this step, microscopic channels were formed in the material which then helped to prevent cracking and deformations during thermal debinding and sintering. Sintering was performed with final temperatures of 700–900 °C. In this temperature range, the shrinkage of the sintered samples scaled linearly with the temperature between 4.4% at 700 °C and 6.6% at 900 °C (Figure 3) and the compressive strength increased from 0.12 to 0.48 MPa, which is at the lower end of compression strengths reported for highly porous Al_2_O_3_ foams [30] and 3D-printed structures [29]. At lower temperatures, the samples were not mechanically stable enough for the handling required in the next steps. At higher temperatures, the silica particles were sintered more densely, leading to a higher shrinkage and mechanical stability, but also to a reduced surface area. Surprisingly, samples sintered at 700 °C were stable enough for further treatment but still showed the same surface area as the starting powder (190 ± 11 m^2^/g; fumed silica powder: 188 ± 12 m^2^/g).

This finding showed that the surface area lost during sintering by joining particles together was either negligible or compensated by an increase in surface area at some point during the fabrication process, for example by breaking up SiO_2_ agglomerates during feedstock preparation. The surface area of samples sintered at 800 and 900 °C was reduced by 7% and 15%, which was unfavorable for the CO_2_ adsorption capacity.

### 3.4. Samples with Different Nozzle Sizes

**Figure 4:**
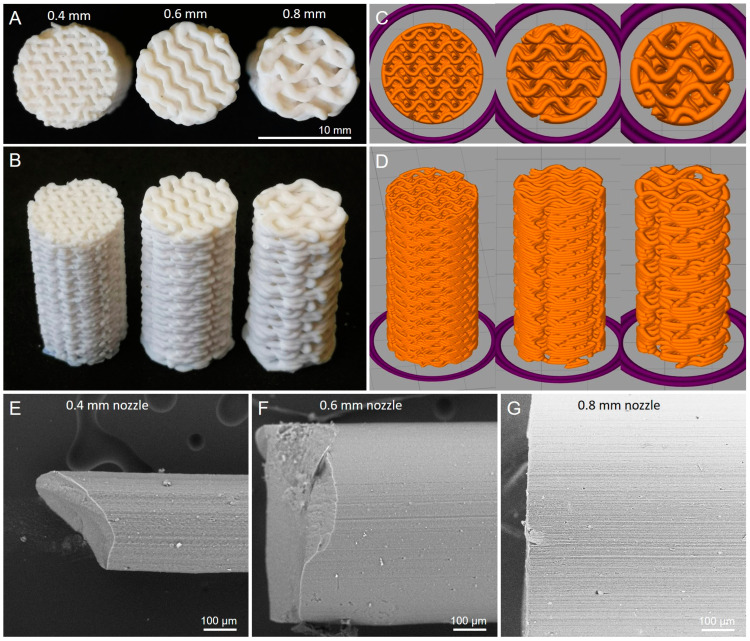
(**A**,**B**) Sintered gyroid cylinders printed with different nozzle sizes. (**C**,**D**) Corresponding slicing patterns. (**E**–**G**) Sintered filament pieces with different diameters.

The resolution of MEX-printed structures is mainly dependent on the printing nozzle diameter. To test the effect of the print resolution on the CO_2_ adsorption performance, cylindrical samples of identical outer dimensions (diameter 10 mm, height 20 mm) were printed with 0.4 mm, 0.6 mm and 0.8 mm nozzles (Figure 4). In each case, the layer height was half of the nozzle diameter. This change affected the unit cell size of the gyroid, increasing it from 2.5 mm (0.4 mm nozzle) to 5.0 mm (0.8 mm nozzle). In all cases, the wall thickness and channel width were set to 120% of the nozzle diameter. Since the infill density was predefined as 50%, the mass of samples printed with different nozzles was approximately 1 g for all samples before debinding and about 0.35 g after sintering.

### 3.5. Filament Granulates

Additionally, the performance (CO_2_ capacity and pressure drop) between 3D-printed monolithic structures and extruded granules was compared. For this, the feedstock was extruded using the same nozzle sizes (0.4–0.8 mm) as for the 3D printing. The extruded filament was then sintered at 700 °C and broken into short pieces (Figure 4E–G).

### 3.6. CO_2_ Capture

Sintered silica samples were functionalized with polyethylenimine (PEI) by applying an ethanolic PEI stock solution (2–10 wt%) to the samples and drying. This functionalization was repeated multiple times to increase the PEI loading and produce samples from 10 to 50 wt% PEI. 

**Figure 5:**
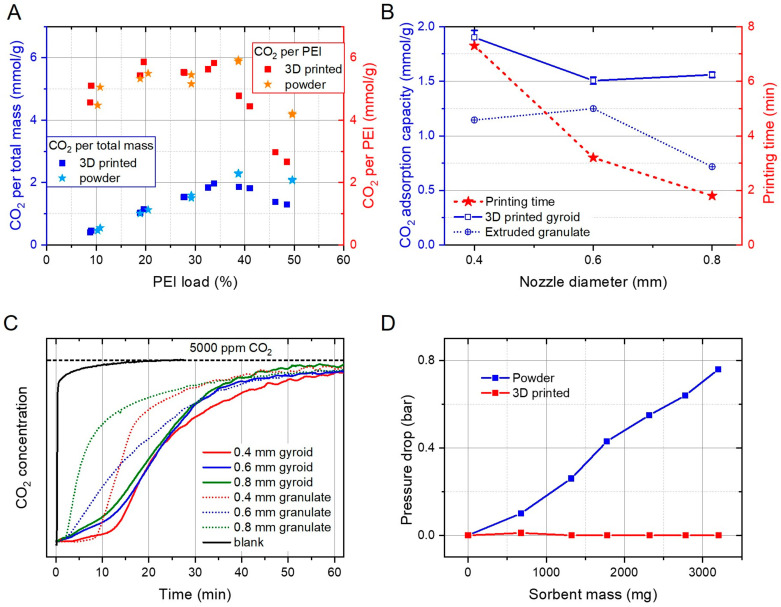
(**A**) CO_2_ adsorption capacity versus PEI load at 25 °C with 5000 ppm CO_2_. CO_2_ capacity per total sorbent mass (substrate + PEI) (left axis, blue) of 3D-printed (square) and powder samples (star). CO_2_ capacity per PEI mass (right axis, red). All samples were printed with a 0.4 mm nozzle. (**B**) CO_2_ adsorption capacity (left axis, blue) for 3D-printed monoliths (squares, solid line) and extruded granules (circles, dotted line) from different nozzle sizes. Printing time for one cylinder of 20 mm height and 10 mm diameter with different nozzle sizes (right axis, red). (**C**) CO_2_ adsorption (5000 ppm of CO_2_ in Ar) on different adsorbents at 25 °C with time. (**D**) Pressure drop across the sorption reactor versus sorbent mass for 3D-printed silica monoliths (red) and silica powder (blue) with a flowrate of 200 mL/min.

CO_2_ adsorption tests showed that the highest CO_2_ capacity in terms of CO_2_ per PEI was achieved between 20 and 34% PEI loading for samples printed with a 0.4 mm nozzle (Figure 5A). At higher loadings, the capacity decreased drastically, indicating that fewer amine groups were accessible for CO_2_ capture because the narrower channels in the support structure were blocked. For the highest efficiency, CO_2_ capacity per total mass (substrate + PEI) should be maximized, and from this perspective the optimal loading was 30–40% PEI, resulting in capacities of 1.9–2.0 mmol/g. At loadings of up to 40 wt% PEI, 3D-printed substrates performed similarly to fumed silica powder. At higher loadings, the accessibility of the PEI functional groups quickly deteriorated while powder samples still performed reasonably well up to 50% PEI. Samples printed with different nozzle sizes and functionalized with 30 wt% PEI showed the highest capacity for 0.4 mm because they had the finest structure and therefore improved accessibility (Figure 5B). The samples printed with 0.6 and 0.8 mm nozzles lost roughly 20% of their capacity due to the larger wall thickness and therefore the reduced accessibility of the amine groups within the structure. On the other hand, the 0.8 mm samples were printed 4 times faster than the 0.4 mm specimens. Alternatively, filaments (0.4–0.8 mm diameter) were extruded, sintered and granulated, then also coated with 30 wt% PEI. Thereof, 0.6 mm diameter granules showed the highest CO_2_ capacity (1.25 mmol/g), which was still substantially less than monolithic substrates for all diameters. This proved that not only the wall thickness of the substrate (as defined by the nozzle size for both monoliths and granules), but also the channel size was crucial for determining the adsorption capacity. 

A comparison of the CO_2_ breakthrough profiles is shown in Figure 5C. The 0.4 mm 3D-printed sample was adsorbing all CO_2_ for roughly 12 min, while 0.6 and 0.8 mm samples leaked some CO_2_ right from the beginning of the experiment due to their larger channels. The same trend was observed with granulated substrates. 

The pressure drop was orders of magnitude smaller for monolithic samples than for powders (Figure 5D). At 3.2 g of substrate, the pressure drop was 0.76 bar for powder, but only <0.01 bar for printed samples. Therefore, the packing height of powdery substrates in the reactor was limited to a few centimeters, but was virtually unlimited for printed samples.

**Figure 6:**
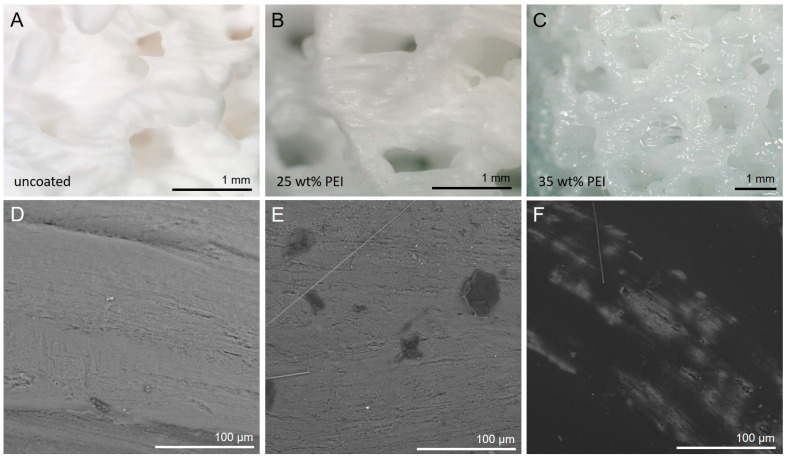
(**A**–**C**) Light microscopy images of 3D-printed substrates with different PEI loading. (**D**–**F**) SEM images of the same substrates with 0, 25 and 35 wt% PEI loading. Silica substrate in grey, PEI chunks in black.

Light microscopy and SEM of samples with different PEI loadings showed that up to approx. 30 wt% PEI was homogeneously incorporated in the silica substrate (Figure 6). At higher PEI loading, the substrate was oversaturated and PEI formed a thick, sticky film on the surface of the substrate, clearly visible by the light reflections. The same effect was observed in SEM: up to 30 wt%, the PEI was virtually invisible because it formed a very thin coating on the substrate. However, at higher PEI loadings, large chunks of PEI agglomerated on the SiO_2_ surface and started to block the pores of the substrate (Figure 6F). This finding explains why there was a maximum CO_2_ adsorption capacity for the 3D-printed structures at around 30–35 wt% PEI loading within this experimental study.

## 4. Conclusions

In summary, a method to 3D-print monolithic structures from fumed silica by MEX 3D printing was presented in this paper. After debinding and sintering, those structures had the same specific surface area as fumed silica powder. The 3D-printed samples were then coated with PEI and the CO_2_ adsorption capacity was measured. The capacities were very similar between 3D-printed and powder substrates, both reaching maximal capacities of 2.0 mmol/g (at 25 °C with 5000 ppm CO_2_) between 30 and 40 wt% PEI loading. The 3D-printed substrates and granules extruded from nozzles with different diameters revealed that finer structures from the smallest nozzle (0.4 mm) were superior in CO_2_ capacity and CO_2_ breakthrough behavior. The main target of this work was to decrease the pressure drop in the adsorption reactor. The results demonstrated a significant reduction in pressure across the 3D-printed sorbents, with measured values of less than 0.01 bar, as compared to 0.76 bar for the equivalent mass of powder sorbent.

## Figures and Tables

**Table 1:** MEX printing conditions.

Nozzle temperature	150 °C
Pre-heating zone temperature	70 °C
Print bed temperature	70 °C
Nozzle Diameter	0.4, 0.6, 0.8 mm
Layer height	Half of the nozzle diameter
Print speed	15 m/s

## Data Availability

The original contributions presented in the study are included in the article, further inquiries can be directed to the corresponding author.
